# Lack of megalin expression in adult human terminal ileum suggests megalin‐independent cubilin/amnionless activity during vitamin B_12_ absorption

**DOI:** 10.14814/phy2.12086

**Published:** 2014-07-22

**Authors:** Louise L. Jensen, Rikke K. Andersen, Henrik Hager, Mette Madsen

**Affiliations:** 1 Department of Biomedicine, University of Aarhus, 8000 Aarhus C., Denmark; 2 Department of Pathology, Aarhus University Hospital, 8000 Aarhus C., Denmark

**Keywords:** Amnionless, cubilin, human terminal ileum, megalin, vitamin B_12_ absorption

## Abstract

Cubilin plays an essential role in terminal ileum and renal proximal tubules during absorption of vitamin B_12_ and ligands from the glomerular ultrafiltrate. Cubilin is coexpressed with amnionless, and cubilin and amnionless are mutually dependent on each other for correct processing to the plasma membrane upon synthesis. Patients with defects in either protein suffer from vitamin B_12_‐malabsorption and in some cases proteinuria. Cubilin lacks a transmembrane region and signals for endocytosis and is dependent on a transmembrane coreceptor during internalization. Amnionless has been shown to be able to mediate internalization of cubilin in a cell‐based model system. Cubilin has additionally been suggested to function together with megalin, and a recent study of megalin‐deficient patients indicates that uptake of cubilin ligands in the kidney is critically dependent on megalin. To further investigate the potential role of amnionless and megalin in relation to cubilin function in terminal ileum and vitamin B_12_ uptake, we initiated a study of *CUBN*/cubilin, *AMN*/amnionless, and *LRP2*/megalin expression in adult human terminal ileum. Our study is the first to reveal the expression pattern of cubilin, amnionless, and megalin in adult human terminal ileum, where cubilin and amnionless localize to the epithelial cells. Surprisingly, we did not detect any megalin protein in adult terminal ileum and consistently, only extremely low amounts of *LRP2 *mRNA. Our data therefore advocate that cubilin and amnionless act independently of megalin in adult terminal ileum and that the cubilin‐megalin interdependency accordingly should be considered as tissue and ligand specific.

## Abstract (precis)

e12086

Studies of human terminal ileum samples demonstrate lack of *LRP2*/megalin expression in adult terminal ileum and point to a megalin‐independent cubilin‐amnionless‐driven uptake mechanism for vitamin B_12_.

## Introduction

Mammals are not able to synthesize vitamin B_12_ (B_12_) but depend on intestinal absorption of dietary B_12_‐supplementations. B_12_ is an essential coenzyme in humans and one classical symptom of B_12_‐deficiency is megaloblastic anemia (Nielsen et al. 2012). B_12_ is absorbed in the terminal ileum bound to the transporter protein intrinsic factor (IF) (Nielsen et al. 2012; Kozyraki and Cases 2013). B_12_ absorption is practically the only function of the terminal ileum other than reabsorption of bile acids, which are absorbed for recycling and transported to the liver via the portal vein (Gourevitch 2005). The IF‐B_12_ complex is suggested to be taken up by the enterocytes via endocytic internalization after binding to the receptor protein cubilin (Fyfe et al. 2004; Andersen et al. 2010; Pedersen et al. 2010; Nielsen et al. 2012). Upon internalization, IF‐B_12_ is released from its receptor; IF is degraded in lysosomes, and B_12_ is delivered to the circulation via the MRP1 transporter protein (Beedholm‐Ebsen et al. 2010).

The ~460 kDa receptor cubilin is expressed in the proximal tubules of the kidney, where it plays a critical role during absorption of filtered proteins, including transferrin (Kozyraki et al. 2001), albumin (Birn et al. 2000; Christensen et al. 2012, 2013; Storm et al. 2013), apolipoprotein A‐I (Kozyraki et al. 1999), and vitamin D‐binding protein (Nykjaer et al. 2001). Cubilin has been shown to bind IF‐B_12_ with high affinity and was for many years considered to be the one and only IF‐B_12_ receptor. When elucidated in the late 1990's, however, its structural organization suggested that one or more additional proteins were required for cubilin to fulfill its role as a receptor (Kozyraki et al. 1998; Moestrup et al. 1998; Kristiansen et al. 1999). The classical structure of a receptor protein includes a transmembrane region, a cytoplasmic tail, and signals for internalization and recycling. Cubilin does not contain any of these features (Kozyraki et al. 1998; Moestrup et al. 1998; Kristiansen et al. 1999). Subsequent genetic studies, performed by Chapelle and coworkers, investigating families with hereditary B_12_‐deficiency, elucidated that mutations in either of the genes encoding cubilin (*CUBN*) (Aminoff et al. 1995, 1999) or amnionless (Tomihara‐Newberger et al. 1998; Kalantry et al. 2001) (*AMN*) (Tanner et al. 2003) can affect B_12_‐absorption, lead to B_12_‐malabsorption and eventually B_12_‐deficiency (Aminoff et al. 1995, 1999; Kristiansen et al. 2000; Tanner et al. 2003). This observation pointed towards a role for amnionless as a coreceptor for cubilin during IF‐B_12_ internalization.

We have previously investigated the proposed coreceptor function of amnionless in a model system with cells expressing recombinant cubilin and amnionless. We showed that the cubilin/amnionless complex (also known as the cubam complex) was indeed able to mediate cellular uptake of IF‐B_12_, and that signals within the cytoplasmic tail of amnionless were sufficient to mediate internalization of cubilin/IF‐B_12_ (Fyfe et al. 2004; Pedersen et al. 2010). Additionally, other cell‐based studies have revealed that cubilin and amnionless are mutually dependent on each other during processing from endoplasmic reticulum, through the Golgi apparatus, and to the plasma membrane. None of the two proteins reaches the plasma membrane if the other protein is absent or malfunctioning (Fyfe et al. 2004; Coudroy et al. 2005).

**Figure 1.**
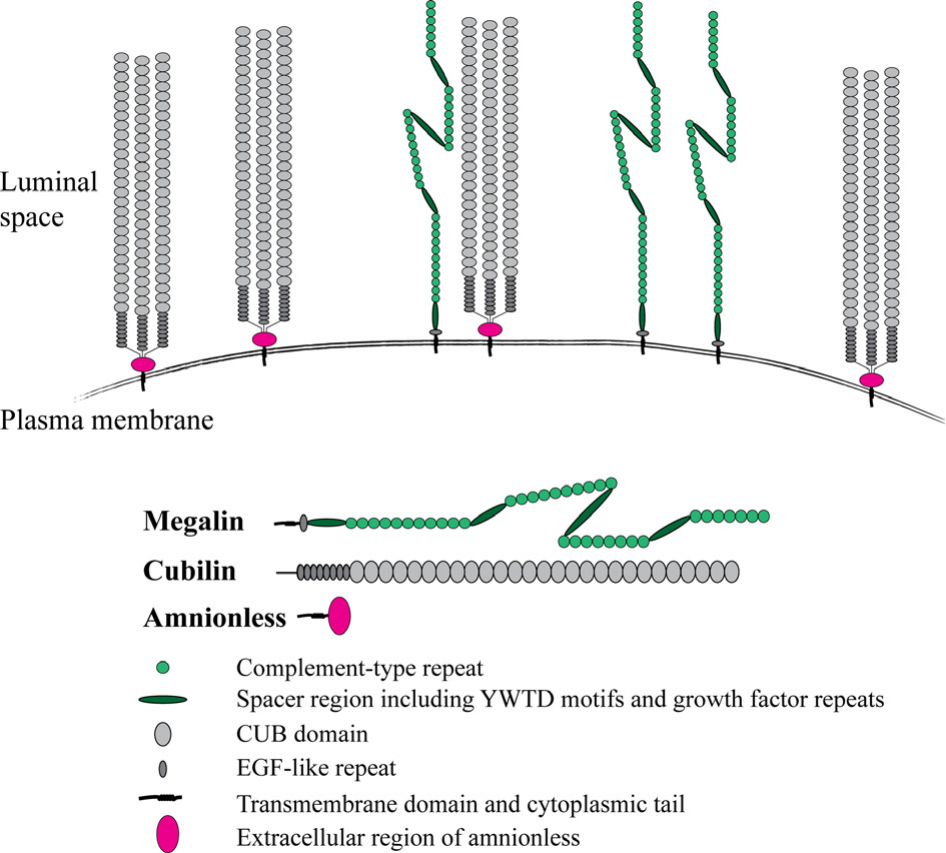
Illustration of the plasma membrane of a cell expressing the receptor protein cubilin and its two suggested co‐receptors: amnionless and megalin. Cubilin, a protein with no transmembrane region, is anchored in the plasma membrane via its interaction with amnionless. The aminoterminal region of cubilin mediates cubilin trimerization and the CUB domains 5–8 bind IF‐B_12_ (Andersen et al. 2010). Amnionless and/or megalin contribute with elements necessary for cubilin‐mediated uptake of ligands. The three proteins are supposedly expressed side by side, but the stoichiometry is not known; neither is it known if cubilin is *always* co‐expressed with both amnionless and megalin.

Long before the identification of the amnionless coreceptor, studies of cubilin characteristics suggested that cubilin function is dependent on the multi‐ligand transmembrane receptor megalin (Birn et al. 1997; Moestrup et al. 1998; Kozyraki et al. 1999). Megalin is coexpressed with cubilin (and amnionless) in kidney proximal tubules and potentially other tissues (as modeled in Fig. 1); megalin can bind to cubilin, and most importantly: megalin deficiency or megalin malfunction results in loss of some cubilin ligands into the urine (Nielsen and Christensen 2010; Christensen et al. 2012, 2013; Storm et al. 2013).

It is known that megalin cannot substitute for amnionless absence or malfunction when it comes to processing of cubilin to the cell surface (Fyfe et al. 2004; Coudroy et al. 2005), but it is unknown if megalin is actively involved during IF‐B_12_ internalization. Important observations suggest the opposite: (1) amnionless can mediate internalization of cubilin‐IF‐B_12_ in a megalin‐free, cell‐based, model system (Fyfe et al. 2004; Pedersen et al. 2010), and (2) patients expressing no or functionally defect megalin do not appear to suffer from B_12_‐malabsorption (Christensen et al. 2013; Storm et al. 2013). Surprisingly, the expression of megalin – or the gene (*LRP2*) encoding it, has until now remained uninvestigated in human terminal ileum.

In this study, we established that *CUBN*,* AMN*, and *LRP2* mRNA expression levels are similar in human kidney cortex. This indicates that cubilin, amnionless and megalin are expressed in similar amounts in the proximal tubules, supporting previous observations. Furthermore, we have investigated the expression of *CUBN*/cubilin, *AMN*/amnionless, and *LRP2*/megalin in adult human terminal ileum. We conclude that ileal *CUBN* and *AMN* are expressed at similar levels. In accordance with this, cubilin and amnionless are both expressed by enterocytes. However, adult terminal ileum lacks expression of *LRP2*/megalin. We therefore propose that intestinal uptake of dietary B_12_ by the cubam complex occurs independently of megalin and that the cubilin‐megalin interdependency accordingly should be considered as tissue and ligand specific.

## Materials and Methods

### Tissue samples and cell lines

Sections of Formalin‐Fixed Paraffin‐Embedded (FFPE), healthy, human terminal ileum biopsies isolated from patients undergoing surgery due to colon cancer (persons of approximately 50–70 years of age) and sections of FFPE, healthy, human kidney were obtained from the pathology archives at Aarhus University Hospital with identifiers removed and in agreement with decision of the Danish ethical committee (Region Midtjylland, DK; case nr. 1‐10‐72‐43‐13). Fresh human terminal ileum and kidney samples were obtained postmortem (and frozen immediately) from deceased persons undergoing autopsy upon family approval to the chief pathologist. A formalin‐preserved biopsy of fetal terminal ileum removed during postmortem autopsy of a deceased 16–20‐week‐old human fetus was obtained upon family approval to the chief pathologist. Total fetal intestinal RNA was purchased from DV Biologics, Costa Mesa, CA, cat. nr.: PD007‐R. A human intestinal cell line, FHs 74 Int (ATCC^®^ CCL‐241™; LCG Standards, Boras, Sweden), was applied in this study. The FHs 74 Int cells were grown in Hybri‐Care Medium (ATCC^®^ 46‐X™) containing 100 units/mL penicillin, 100 *μ*g/mL streptomycin, and supplemented with 30 ng/mL epidermal growth factor (Sigma, Copenhagen, Denmark, cat. nr.: E9644) and 10% fetal calf serum.

### RNA purification

RNA was purified from frozen tissue using the RNeasy Mini Kit (Qiagen, Copenhagen, Denmark) according to manufacturer's instructions. For each batch of purification, a maximum of 30 mg of tissue was homogenized at 25 Hz for 3 min using a Tissuelyser II machine (Qiagen) and 5 mm Stainless Steel Beads (Qiagen). Homogenized tissue was purified using RNeasy Mini columns, and subsequent to purification, on‐column DNase digestion of the RNA for elimination of DNA contamination was performed (RNase‐Free DNase Set, Qiagen). RNA was purified from FFPE samples/formalin‐fixed tissues using the RNeasy FFPE Kit (Qiagen). Five sections of 10 *μ*m were pooled and deparaffinated in deparaffination Solution (Qiagen) and RNA was subsequently purified according to manufacturer's instructions for optimized removal of genomic DNA contamination. Quantification of total RNA was measured using a Thermo Scientific NanoDrop 1000™ (Thermo Scientific, Hvidovre, Denmark) and Thermo Scientific Software ND.1000 V3.5.2.

### One step reverse transcription‐polymerase chain reaction

Reverse Transcription‐Polymerase Chain Reaction (RT‐PCR) was performed using the One‐Step RT‐PCR Kit (Qiagen) according to manufacturer's instructions. Primers recognizing the genes of interest were designed according to standard parameters using primer3 (http://primer3.wi.mit.edu/) and Primer‐BLAST (www.ncbi.nlm.nih.gov/tools/primer-blast/) and synthesized at Tag Copenhagen A/S (Copenhagen, Denmark). For *CUBN* and *LRP2*, additional short amplification product primers were applied for reactions with FFPE‐extracted RNA. A complete list of all primers used in this study is shown in Table 1. For each RT‐PCR reaction the following components were included in the reaction mixture: 100–500 ng total human terminal ileum and/or kidney RNA (the amount of template in the different experiments are given in the figure legends), gene‐specific primers, dNTPs, Qiagen One‐Step RT‐PCR buffer, Q‐solution for amplifying *AMN* PCR products, which enables efficient amplification of GC‐rich templates, and Qiagen One‐Step RT‐PCR enzyme mix. The PCR cycling parameters were as follows: 94°C for 15 min for enzyme activation, 45 cycles of 94°C for 60 s, 60°C for 60 s, and 72°C for 90 s, and finally one cycle of 72°C for 10 min. PCR amplification products were analyzed by 2% agarose gel electrophoresis and visualized using SYBRgreen and a Fuji Film LAS 3000, using Image Reader LAS‐3000 software, Version 2.2 (Science Imaging Scandinavia AB, Saltsjö‐Boo, Sweden).

**Table 1.** List of primers used for RT‐PCR and Taqman assays used for qPCR. Primers denoted ‘Short’ were used for RT‐PCR amplification using RNA isolated from FFPE tissue samples as template.

Name	Sequence	Reference ID	Exon boundary^1^	Location (nt)^1^	Amplicon length (bp)
Primers for RT‐PCR					
* hGAPDH_FW*	5′‐CGACAGTCAGCCGCATCTT‐3′	NM_002046.5	1–2	132–194	63
*hGAPDH_R*	5′‐CCCCATGGTGTCTGAGCG‐3′
h*CUBN*_FW	5′‐AATGGATGTGTGCAGCTCAG‐3′	NM_001081.3	11–12	1220–1372	153
*hCUBN_R*	5′‐GGGGTTGCTCAAACACTCAT‐3′				
*hLRP2_FW*	5′‐TGAAATTGGCTG CGCTGTTGTGACC‐3′	NM_004525.2	2–4	387–624	238
*hLRP2_R*	5′‐AG CTCCATCGGGG CAGTCTCTG‐3′				
h*AMN*_FW	5′‐GTCTCCAAACTCTGGGTCC‐3′	NM_030943.3	2–3	91–206	116
*hAMN_R*	5′‐ACTGACACCATCTTGTCCG‐3′				
h*CUBN*short_FW	5′‐TATGGAAGAGTGTGGTGG‐3′	NM_001081.3	47–48	7396–7466	71
h*CUBN*short_R	5′‐TTGGGTTCGGGTAGTTGG‐3′				
h*LRP2*_short_FW	5′‐GTTGACCTGAAACTGAAATAC‐3′	NM_004525.2	67–68	12613–12683	71
h*LRP2*_short_R	5′‐TAAATATGCCTTCCAACCC‐3′				

Target gene	Taqman assay ID	Reference ID	Exon boundary^1^	Assay location^1^	Amplicon length (bp)
Taqman assays for qPCR					
*GAPDH*	Hs02758991_g1	NM_002046.5	7–8	704	93
*ACTB*	Hs01060665_g1	NM_001101.3	2–3	208	63
*RPLP0*	Hs04189669_g1	NM_053275.3	1–2	129	65
*CUBN*	Hs00153607_m1	NM_001081.3	43–44	6699	92
*AMN*	Hs00229558_m1	NM_030943.3	3–4	240	110
*LRP2*	Hs00189742_m1	NM_004525.2	34–35	5862	72

H, human; GAPDH, glyceraldehyde 3‐phosphate dehydrogenase; CUBN, cubilin; LRP2, low‐density lipoprotein receptor‐related protein 2; AMN, amnionless; ACTB, beta actin; RPLP0, large ribosomal protein P0; nt, nucleotides; bp, base pairs.

^1^ According to the NCBI reference sequence. Reference ID sequences represent cDNAs.

### Generation of cDNA

RNA was reverse transcribed to cDNA using the High Capacity RNA‐to‐cDNA Kit (Applied Biosystems, Life Technologies, Naerum, Denmark), according to manufacturer's instructions. Each reaction mixture included the follwing: 10 *μ*L 2× RT Buffer, 1 *μ*L Enzym Mix, 250 ng–1.5 *μ*g RNA template (dependent of the amount and concentration of RNA available), and RNase‐free water to a total reaction volume of 20 *μ*L. Minus Reverse transcriptase (−RT) reactions (enzyme‐free) were performed in connection to each tissue sample. All variables (procedure, conditions, and concentration of template) were identical within each separate study.

### Quantitative real‐time PCR (qPCR)

The following TaqMan^®^ gene expression assays (Applied Biosystems) were applied: *CUBN* (Hs00153607_m1), *AMN* (Hs00229558_m1), *LRP2* (Hs00189742_m1), *ACTB* (Hs01060665_g1), *RPLP0* (Hs04189669_g1), and *GAPDH* (Hs02758991_g1). qPCR was performed using an Applied Biosystems 7500 fast qPCR machine according to standard procedures. All reactions (except the NTC control reactions) included the following: 1 *μ*L 20× TaqMan^®^ Gene Expression Assay, 10 *μ*L TaqMan^®^ Fast Advanced Master Mix, 4 *μ*L cDNA template, and 5 *μ*L RNase‐free water. Each sample was run in triplicate for each TaqMan^®^ assay.

### Calculation of relative expression levels

qPCR data were analyzed using 7500 Software v2.0.6, Applied Biosystems and Microsoft Office Excel 2007. Normalization of obtained *C*_t_ values (Δ*C*_t_ values) was calculated using *ACTB, GAPDH* (not shown), and *RPLP0* (not shown) as reference genes. Δ*C*_t_ values were calculated as *C*_t_ (target)‐*C*_t_ (*normalizing gene*). All qPCR experiments and calculations were performed in accordance with the MIQE guidelines (Bustin et al. 2009).

### Statistics

For statistical calculations, we used Sigma Stat 10.0. Results in this paper are reported as Δ*C*_t_ ± standard error of mean (SEM). Normalized *C*_t_‐values (Δ*C*_t_ values) were compared by Student's *T*‐test. *P* < 0.05 was considered as statistically significant.

### Immunohistochemistry

Paraffin‐embedded blocks of human ileum tissue was cut into 3 *μ*m‐thick sections, mounted on Superfrost^®^ glas (Thermo scientific) and dried for 1 h at 60°C. The sections were deparaffinated in Tissue‐Clear, rehydrated in graded alcohol, and washed in distilled water. Endogenous peroxidase activity was blocked by EnVision™ FLEX+ Peroxidase Blocking agent (DAKO, Copenhagen, Denmark) for 5 min. Epitope retrieval was performed by microwave heating for six cycles: 8 min at 800 W and 2 × 14 min at 480 W in Tris/EGTA retrieval buffer, pH 9. Antigen–antibody binding was performed at room temperature for 30 min. Each incubation step was followed by washes in TBS buffer (Tris‐buffered saline: 50 mmol/L Tris, 150 mmol/L NaCl, with 0.05% Tween 20, pH = 7.6). The primary antibodies used were as follows: polyclonal rabbit anti‐human megalin antibody [generous gift from prof. Dr. S. K. Moestrup, Aarhus University, Aarhus, Denmark, used in (Prabakaran et al. 2011)], polyclonal rabbit anti‐human cubilin antibody [generous gift from prof. Dr. S. K. Moestrup, Aarhus University, Aarhus, Denmark, used in (Prabakaran et al. 2012)] and goat anti‐human amnionless antibody (R&D Systems Europe, Oxon, UK, cat. nr.: AF1860). EnVision™+ Dual Link System‐HRP (DAKO) and 3, 3‐diaminobenzidine tetrahydro‐chloride (Kem‐En‐Tek Nordic, Taastrup, Denmark) was used as secondary reagent and for visualization, respectively. In case of the goat anti‐human amnionless antibody, a rabbit‐anti goat linker (P0160, DAKO) was applied prior to the secondary reagent. The reactions were enhanced with 0.5% CuSO_4_. The sections were counterstained with Mayer's hematoxylin. Finally, the sections were dehydrated and mounted with Pertex (Histolab, Göteborg, Sweden). Sections were analyzed using a light microscope (Nikon Eclipse 55i; DFA instruments, Glostrup, Denmark).

## Results

### Quantitative mRNA analyses indicate that *CUBN*,* AMN*, and *LRP2* are expressed at similar levels in human kidney cortex

The interplay between cubilin and its two suggested coreceptors: amnionless and megalin, is well characterized in the kidney. Missing expression of amnionless or amnionless malfunction in the proximal tubules results in retention of cubilin within the endoplasmic reticulum, absent membrane localization, cubilin malfunction, and consequently, loss of cubilin ligands with the urine (He et al. 2005). Megalin malfunction or missing megalin expression impairs proximal tubule reabsorption of *some* cubilin ligands, but not all (Storm et al. 2013), indicating a ligand‐specific dependency. Despite the evident cooperativity of these three proteins in human proximal tubule cells, their relative expression levels have not been quantitatively explored.

**Figure 2.**
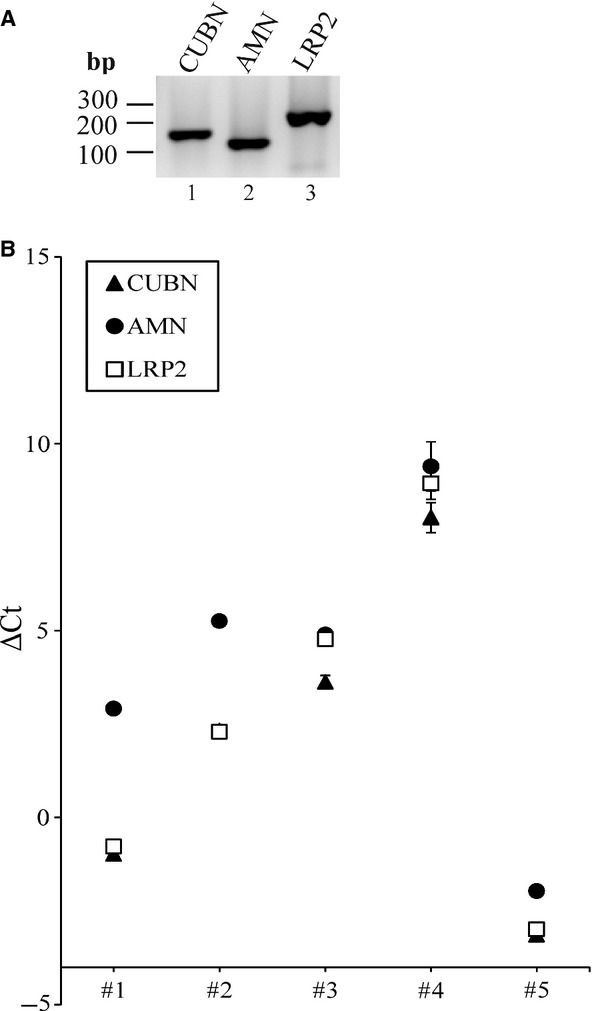
Characterization of the levels of mRNA encoding cubilin, amnionless and megalin in human kidney cortex. (A) Representative RT‐PCR products obtained using RNA isolated from a random human kidney cortex biopsy. Amplification with primers encoding products of *CUBN*,* AMN*, and *LRP2*; 500 ng total RNA was used as template in each reaction. Lane 1: *CUBN *= 153 bp, lane 2: *AMN *= 116 bp, lane 3: *LRP2 *=**238 bp. Products were analyzed on a 2% agarose gel and visualized using SybrGreen. (B) qPCR: Normalized *C*_t_ values (Δ*C*_t_ values) of *CUBN*,* AMN* and *LRP2*. *ACTB* was used as normalizing gene. *C*_t_ values were obtained by qPCR using cDNA generated from RNA isolated from human kidney cortex. In total, samples from five different human kidneys were analyzed, each in nine replicates.

We analyzed the presence of mRNA encoding cubilin, amnionless and megalin in RNA samples isolated from fresh human kidney cortex (isolated postmortem during autopsy) by semiquantitative RT‐PCR analyses. RNA from 5 different kidneys was analyzed. Representative data from one kidney are shown in Fig. 2A. We detected PCR products encoding *CUBN*,* AMN*, and *LRP2* of the expected sizes of 153 bp, 116 bp, and 238 bp, respectively. An additional band was observed in the *LRP2*‐lane (Fig. 2A, lane 3). This band was identified as primer dimer. All product identities were verified by sequencing. The levels of mRNA were explored quantitatively by qPCR, and normalized *C*_t_‐values (Δ*C*_t_‐values) of *CUBN, AMN,* and *LRP2* are shown in Fig. 2B. *CUBN* Δ*C*_t_ varied from −3.13(SEM ± 0.11) to 8.03(SEM ± 0.85); *AMN* Δ*C*_t_ varied from −1.96(SEM ± 0.058) to 9.40(SEM ± 0.65); and *LRP2* Δ*C*_t_ varied from −2.99(SEM ± 0.043) to 8.94(SEM ± 0.42). *ACTB* was used as normalizing gene. Five different human kidney cortex samples were analyzed in nine replicates each. We found no statistical significant difference between the Δ*C*_t_‐values of *CUBN, AMN,* and *LRP2* mRNA levels measured in these five human kidney cortex samples. This indicates similar expression levels of *CUBN*,* AMN*, and *LRP2* in kidney proximal tubules in accordance with their interdependency and cofunction during reabsorption of ligands from the glomerular ultrafiltrate.

### *CUBN* and *AMN* mRNAs are expressed at similar levels in adult human terminal ileum while adult human ileal *LRP2* expression is extremely low

Despite speculations regarding cubilin‐amnionless‐megalin cofunction in human terminal ileum, their expression pattern in tissue samples of *human* terminal ileum has never been examined, neither at mRNA nor protein level.

**Figure 3.**
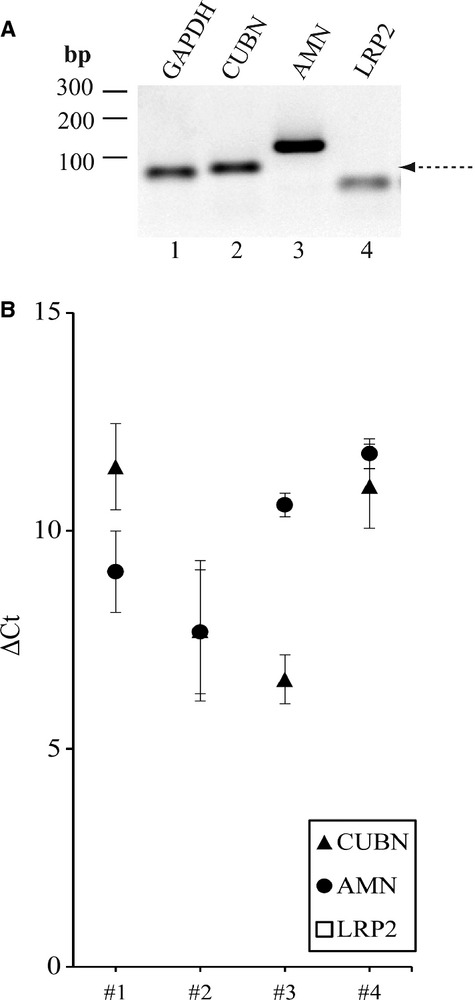
Characterization of the levels of mRNA encoding cubilin, amnionless and megalin in human terminal ileum. (A) Representative RT‐PCR products obtained using RNA isolated from a random human terminal ileum FFPE biopsy. Amplification was performed with primers encoding: *CUBN*,* AMN*,* LRP2,* and *GAPDH* (endogenous control) Lane 1: *GAPDH *= 62 bp, lane 2: *CUBN *= 71 bp, lane 3: *AMN *= 116 bp, lane 4: primer dimers ≈ 50 bp (no band of the expected size of 71 bp for *LRP2*). (B) qPCR: Normalized *C*_t_ values (Δ*C*_t_ values) of *CUBN*,* AMN* and *LRP2*. *ACTB* was used as normalizing gene. (*n *= 4, each sample analyzed in 3 separate experiments, each experiment performed in triplicates).

Presence of mRNA encoding ileal cubilin, amnionless and megalin was analyzed by RT‐PCR in sections of four different FFPE biopsies of adult human terminal ileum. Primers were designed for the amplification of relatively small products (of ~100 base pairs or less) to account for the risk of RNA degradation during processing of paraffin‐embedded tissue samples. Representative *CUBN* and *AMN* PCR products (of 71 bp and 116 bp, respectively) are shown in Fig. 3A (lane 2–3) along with the product of *GAPDH* (lane 1) (products were verified by sequencing, template concentration was 100 ng in all reactions). However, the expected *LRP2* PCR product of 71 bp (indicated with an arrow in lane 4) could not be obtained – only ~50 bp primer‐dimers. The applied *LRP2* primer pair was tested fully functional and specific using kidney cortex RNA as template (not shown here). Use of alternative *LRP2* primers or increasing the template concentration did not alter the result; *LRP2* products from all ileum RNA samples analyzed were either absent or extremely faint.

To confirm this pattern and furthermore investigate the mRNA levels quantitatively, we investigated the levels of mRNA encoding cubilin, amnionless, and megalin in the four above mentioned adult human terminal ileum FFPE biopsies by qPCR (Fig. 3B). The *CUBN*,* AMN* and *LRP2* mRNA levels (in terms of *C*_t_‐values) were measured and normalized to the *C*_t_‐value of *ACTB*. The normalized *C*_t_‐values (Δ*C*_t_) for *CUBN*,* AMN*, and *LRP2* are shown in Fig. 3B. The *CUBN* Δ*C*_t_ values varied from 6.59(SEM ± 0.56) to 11.47(SEM ± 0.99), and the *AMN* Δ*C*_t_ values varied from 7.68(SEM ± 1.42) to 11.77(SEM ± 0.34). We thus found no statistical significant difference between the *CUBN* mRNA level and the *AMN* mRNA level in these four human terminal ileum biopsies, indicating that *CUBN* and *AMN* are expressed at similar level in adult human terminal ileum. *LRP2* mRNA encoding megalin was undetectable in these four samples and therefore no Δ*C*_t_ value could be calculated (“*LRP2*” markings are absent in Fig. 3B). Thus, both qPCR and RT‐PCR data indicate absent or very low *LRP2* mRNA levels in the ileal biopsies tested here and presumably in adult human terminal ileum in general.

### Pairwise analyses of *CUBN*,* AMN*, and *LRP2* mRNA levels in fresh‐frozen samples of adult human terminal ileum and kidney cortex confirms comparable levels of *CUBN* and *AMN* mRNA and low *LRP2* expression in adult terminal ileum

**Figure 4.**
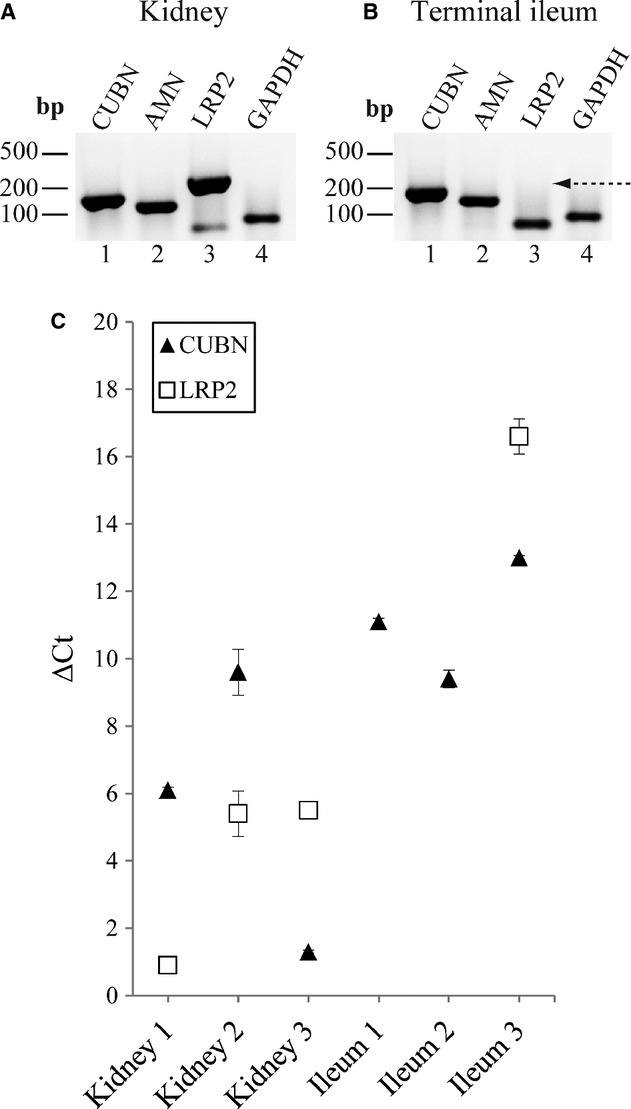
Pairwise analyses of *CUBN*,* AMN*, and *LRP2* mRNA levels in fresh‐frozen samples of human terminal ileum and kidney cortex by RT‐PCR and qPCR. RT‐PCR amplification of RNA with primers encoding products of: *CUBN*,* AMN*,* LRP2,* and *GAPDH*; in (A) Human kidney, and (B) Terminal ileum. About 500 ng total RNA was used as template in each reaction. Lane 1: *CUBN *= 153 bp, lane 2: *AMN *= 116 bp, lane 3: *LRP2 *=**238 bp, lane 4: *GAPDH *= 63 bp. In panel B, lane 3, only primer dimers of ~ 50 bp could be observed (no band of the expected size of 238 bp for *LRP2*). *GAPDH* was used as endogenous control gene. Products were analyzed on a 2% agarose gel and visualized using SybrGreen. (C) Pairs of biopsies of human terminal ileum and kidney cortex of three different individuals were analyzed. Normalized *C*_t_ values (Δ*C*_t_ values) of *CUBN* and *LRP2* obtained analyzing the three kidney cortex and the three ileum samples, respectively. In the terminal ileum samples, *LRP2* products were undetectable in two out of three samples, and only one *C*_t_ value was measured. *ACTB* was used as reference gene in all qPCR (each sample analyzed in three separate experiments, each experiment performed as triplicates).

To validate our data indicating presence of mRNA encoding cubilin and amnionless, but absence or low levels of mRNA encoding megalin in adult human terminal ileum, we obtained pairs of fresh‐frozen samples of terminal ileum and kidney cortex isolated from the same person (postmortem; during autopsy) to account for an ‘overall’ megalin‐deficiency and possible differences in tissue preparation. We included samples from three individuals. The levels of mRNA encoding cubilin and megalin in kidney cortex and terminal ileum were measured by RT‐PCR (Fig. 4A–B) and by qPCR (Fig. 4C). In Fig. 4; representative RT‐PCR products from one patient's kidney cortex *and* terminal ileum are shown. PCR products encoding *CUBN*,* AMN,* and *LRP2* of the expected sizes of 153 bp, 116 bp and 238 bp, respectively, were amplified successfully from kidney cortex RNA (Fig. 4A, lane 1–3, respectively). From terminal ileum RNA, only *CUBN* and *AMN* (along with *GAPDH*) PCR products of the expected size and sequence could be amplified (Fig. 4B, lanes 1–2 and 4). No *LRP2* PCR product of the expected 238 bp size was amplified from terminal ileum RNA (Fig. 4B, lane 3) (indicated with an arrow), only a product of ~ 50 bp (primer‐dimer). Use of alternative primer pairs for *LRP2* did not change the result. The RNA isolated pairwise from kidney cortex and terminal ileum was further analyzed by qPCR (Fig. 4C). All measured *C*_t_‐values were normalized to the *C*_t_‐value of *ACTB*. Normalized *C*_t_ values (Δ*C*_t_ values) of *CUBN* in kidney varied from 1.37(SEM ± 0.047) to 9.57(SEM ± 0.68) and in terminal ileum from 9.40(SEM ± 0.26) to 11.1(SEM ± 0.10) (not statistically significantly different; *P* = 0.052). Δ*C*_t_ values of *LRP2* in kidney varied from 0.92(SEM ± 0.088) to 5.49(SEM ± 0.079) (confirming similar levels of *CUBN* and *LRP2* mRNA in kidney cortex). On the other hand, *LRP2* products were only detected in one of three ileum samples. The single calculable Δ*C*_t_ value for *LRP2* in terminal ileum was of 16.62(SEM ± 0.52), indicating >2000 fold lower expression of *LRP2* in ileum compared to kidney from the same patient. The considerable difference in tissue expression of *LRP2* indicates that this receptor plays a less significant or much different role in adult terminal ileum than it does in kidney cortex.

### Immunohistochemical analyses of sections of paraffin‐embedded adult human terminal ileum confirms expression of cubilin and amnionless at protein level and demonstrate absent ileal expression of megalin

**Figure 5.**
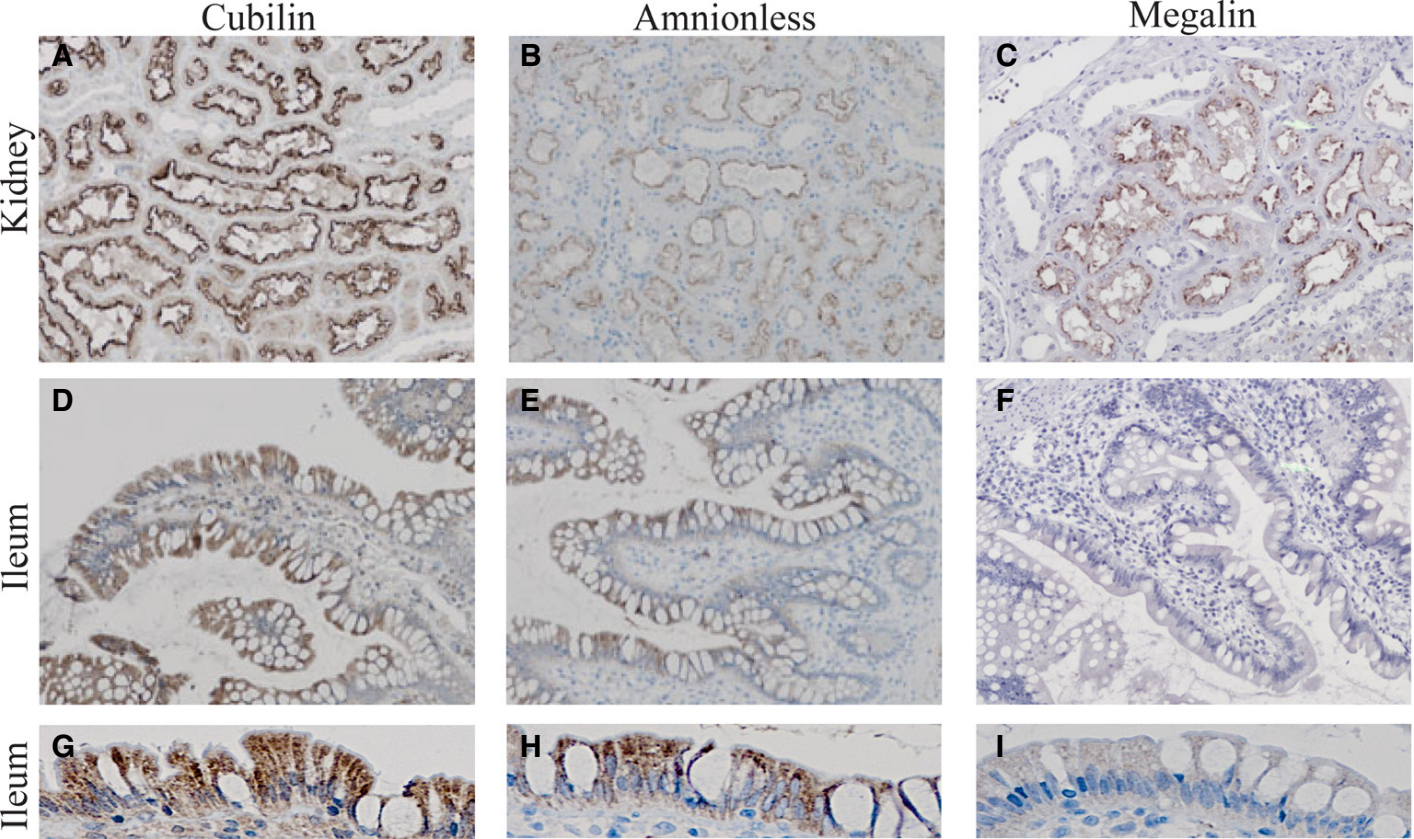
Visualization of the cubilin, amnionless, and megalin protein expression in human terminal ileum. Labeling of cubilin, amnionless, and megalin protein in paraffin sections of kidney cortex showed the expected expression in the proximal tubules of all three proteins and confirmed antibody specificity and functionality (panels A–C). Sections of a paraffin‐embedded biopsy of human terminal ileum were stained positive for expression of cubilin (D) and amnionless (E) protein, whereas megalin protein was undetectable in this ileum sample (F). The stainings were performed by the use of (A,D) polyclonal rabbit anti‐human cubilin antibody, titer 1:100; (B,E) polyclonal goat anti‐human amnionless antibody, titer 1:200; and (C,F) polyclonal rabbit anti‐human megalin antibody, titer 1:100. Images G–I represent images at higher magnification of the stained sections presented in D–F.

With the indication that only cubilin and amnionless are expressed in adult human terminal ileum, we investigated the expression of cubilin, amnionless, and megalin at protein level by immunohistochemistry. Sections of FFPE human terminal ileum biopsies were stained using polyclonal antibodies generated against full length human cubilin, the extracellular region of human amnionless and full length human megalin, respectively. A series of stained sections of a representative human terminal ileum biopsy is shown in Fig. 5D–F. Human kidney was included as positive control using the same antibodies and same procedure (Fig. 5A–C). Both ileal cubilin and amnionless protein was detectable in the polarized epithelial cells lining the lumen, indicating coexpression and colocalization (Fig. 5D–E). Megalin protein expression was, as expected, not detectable above background level (Fig. 5F). Images of ileal sections stained for cubilin, amnionless and megalin at higher magnification are presented in Fig. 5G–I. They confirm that the enterocytes clearly express cubilin and amnionless, but no megalin.

Our study is the first to show expression of *CUBN*/cubilin and *AMN*/amnionless mRNA and protein in adult human terminal ileum. Our results furthermore demonstrate lack of megalin expression in adult terminal ileum. Our data therefore point to a tissue‐specific cooperative receptor relation between the cubilin/amnionless complex and megalin.

### Analyses of human fetal terminal ileum and samples of total fetal small intestine indicate ileal expression of *LRP2* during fetal life

**Figure 6.**
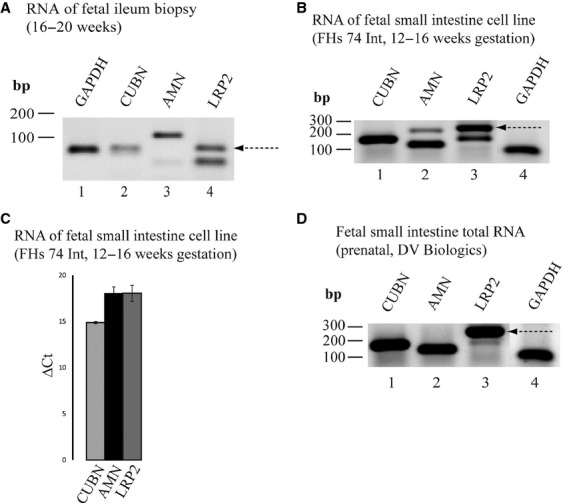
Characterization of the levels of mRNA encoding cubilin, amnionless and megalin in human fetal small intestine including terminal ileum. (A) RT‐PCR using RNA isolated from a human fetal terminal ileum biopsy (16–20 weeks gestation). Lane 1: *GAPDH* = 63 bp, lane 2: *CUBN *= 71 bp, lane 3: *AMN *= 116 bp, and lane 4: *LRP2*: 71 bp and primer dimers ~ 50 bp. (B) RT‐PCR using RNA isolated from a cell line generated from human fetal small intestine. Lane 1: *CUBN *= 153 bp, lane 2: *AMN *= 116 bp, lane 3: *LRP2 *=**238 bp, lane 4: *GAPDH* = 63 bp. (C) qPCR: qPCR using cDNA generated from RNA isolated from a cell line generated from human fetal small intestine. (D) RT‐PCR using a commercially available total RNA pool purified from human fetal small intestine. Lane 1: *CUBN *= 153 bp, lane 2: *AMN *= 116 bp, lane 3: *LRP2 *=**238 bp, lane 4: *GAPDH* = 63 bp. All PCR products were analyzed on a 2% agarose gel and visualized using SybrGreen.

The data presented above were obtained using adult human ileal material. We furthermore explored the expression of *CUBN*,* AMN,* and *LRP2* mRNA in a formalin‐preserved biopsy of terminal ileum removed during postmortem autopsy of a deceased 16–20‐week‐old human fetus. As shown in Fig. 6A, we were able to detect *CUBN*,* AMN,* and *LRP2* RT‐PCR products of the expected sizes (71 bp, 116 bp, and 71 bp, respectively) within RNA of this biopsy. Primer dimer products of the *LRP2*‐primer set were produced in addition to the specific PCR product (lane 4) indicating low template abundance. We confirmed fetal intestinal *LRP2* mRNA expression in a commercially available fetal intestinal cell line (human fetus; 16–20‐weeks‐old) (Fig. 6B; products of 153 bp, 116 bp, and 238 bp, respectively; additional bands in the *AMN*‐ and *LRP2*‐lanes were sequenced and shown to be non‐specific PCR products). We also confirmed fetal intestinal *LRP2* mRNA expression in a commercially available pool of total RNA isolated from fetal small intestine (Fig. 6D). The relative levels of mRNA encoding cubilin, amnionless and megalin in the fetal small intestine‐derived cell line were additionally measured by qPCR (Fig. 6C), indicating comparable levels of *AMN* and *LRP2* mRNA expression in fetal small intestine.

Based on these results the *LRP2*/megalin expression in human terminal ileum appears to differ between the fetal and adult developmental stage. Our data indicate that *CUBN*,* AMN,* and *LRP2* mRNA is expressed in fetal small intestine at comparable levels (Fig. 6); that *CUBN*/cubilin and *AMN*/amnionless expression is maintained in human terminal ileum in adult life at mRNA and protein level (Figs. 3–5), and that *LRP2* expression in human terminal ileum decreases to insignificant and practically undetectable levels from fetal to adult life.

## Discussion

Cubilin and megalin have been suggested to cofunction in a number of polarized, absorptive tissues, including kidney cortex, ileum, and yolk sac. Various studies throughout the past ~25 years have studied these two multi‐ligand receptor proteins and their individual and combined functions. It is evident that cubilin is dependent on megalin to fulfill its role during absorption of ligands from the glomerular ultrafiltrate in the kidney proximal tubules. Some cubilin ligands are lost with the urine if megalin function is impaired or if megalin is absent, most recently reported in studies of patients with Donnai‐Barrow syndrome (Storm et al. 2013). Whether the interplay between megalin and cubilin is direct or indirect has not yet been clarified, but it is known that megalin can bind cubilin with relatively high affinity (Moestrup et al. 1998). It is, however, not known if they actually interact in vivo. In line with the evident requirement for megalin during absorption of certain cubilin ligands, we investigated the levels of mRNA encoding cubilin, amnionless, and megalin in kidney cortex to establish their relative expression levels. The results of our analyses revealed that *CUBN*,* AMN,* and *LRP2* mRNAs are expressed at similar levels. This is in accordance with a number of studies reporting that RAP‐affinity chromatographic analyses of solubilized kidney cortex membranes results in purification of similar amounts of cubilin and megalin (24) and that amnionless is copurified with cubilin (Fyfe et al. 2004).

Peculiarly, only rescue of *some* cubilin ligands from the glomerular ultrafiltrate is impaired in case of megalin‐deficiency (Christensen et al. 2013; Storm et al. 2013). Furthermore, there are no indications that megalin‐deficient patients suffer form B_12_‐malabsorption (Storm et al. 2013). This points towards a megalin‐independent uptake‐mechanism for B_12_ in terminal ileum. In line with this, we have previously shown that cubilin and amnionless expression is sufficient to ensure uptake of iodinated IF‐B_12_ in a cell‐based model system expressing no megalin (Fyfe et al. 2004; Pedersen et al. 2010). In this study, we therefore questioned the long‐standing assumption that megalin functions as coreceptor during uptake of *all* cubilin ligands, and specifically, if megalin is involved in ileal uptake of dietary B_12_. To enlighten this, we investigated the expression of *CUBN*/cubilin, *AMN*/amnionless, and *LRP2*/megalin in adult *human* terminal ileum. Our results indicate that *CUBN* and *AMN* mRNA levels in adult human terminal ileum are similar (Fig. 3), supporting their cofunction as a receptor complex for IF‐B_12_, ensuring dietary B_12_‐uptake. Surprisingly*, LRP2* mRNA was undetectable by RT‐PCR and qPCR in adult human terminal ileum. We confirmed this result by comparison of pairs of samples of kidney cortex and terminal ileum, each pair of kidney and ileum sample collected from the same individual to account for any case of inherent megalin‐malfunction or ‐deficiency. *CUBN* and *AMN* mRNA levels were again similar, and *LRP2* mRNA was either absent or detectable in minute amounts, several magnitudes below *CUBN* and *AMN* mRNA levels. We additionally investigated cubilin, amnionless and megalin protein expression in adult human terminal ileum. As expected, we detected both cubilin and amnionless protein expression in the epithelial cells of terminal ileum, while no megalin protein was detectable.

Combined, our data therefore suggest that epithelial cells of adult terminal ileum co‐express cubilin and amnionless and that the cubam complex functions independently of megalin during IF‐B_12_ uptake. Our data indicate that megalin does not serve the same coreceptor role to cubilin in terminal ileum as it does in the renal proximal tubules. We therefore suggest that previous models designating megalin as an obligatory coreceptor for cubilin should be revised according to our results. Data of other studies also point towards a tissue‐specific interdependency between cubilin and its two partners; amnionless and megalin. For example, from studies of fetal mouse development, it was reported that amnionless is expressed in neuroepithelium at hardly detectable levels, and that it is not expressed in cephalic neural crest cells at all; sites which have both been shown to express cubilin and megalin and play a crucial role during mouse brain development (Cases et al. 2013). Based on its hitherto described co‐function with cubilin, megalin was accordingly suggested to support cubilin function in these expression sites instead of amnionless and specifically to mediate internalization of cubilin and associated ligands (Cases et al. 2013).

Due to differences in implications on fetal mouse development originating from silencing *cubn* or *lrp2*, it has furthermore been suggested that an additional third and yet unidentified partner of cubilin may exist in the fetal neuroepithelium (Cases et al. 2013). This may of course also be the case in adult terminal ileum. From our studies, it is evident that megalin cannot support cubilin function in adult terminal ileum; on the other hand, we cannot rule out that cubilin interacts with another receptor besides amnionless.

Our results contradict with the results of previous studies reporting expression of *LRP2*/megalin in terminal ileum (Birn et al. 1997; He et al. 2005). These studies were all, however, performed using tissue samples of animal origin. In addition, the animal tissue samples used in the previous studies were collected from fairly young animals, whereas the human samples applied in the experiments described above were collected from adult human beings. By using human fetal tissue samples or human cells of fetal origin, we too were able to detect expression of *LRP2* mRNA in the small intestine, including terminal ileum. The expression of *LRP2* in the analyzed sample of fetal terminal ileum appeared to be low, which was apparent from the low yield of our *LRP2*‐RT‐PCR and the amplification of non‐specific RT‐PCR products. The yield of the *CUBN* and *AMN* reactions using this sample were quite low as well, which could indicate poor preservation of the RNA in this biopsy. It is therefore difficult to judge if the *LRP2* mRNA expression is significantly lower than *CUBN* and *AMN* mRNA expression levels. We were not able to confirm the expression of *LRP2* at protein level in a fetal terminal ileum biopsy due to limited sample amount and availability. We could not demonstrate megalin expression in cultured cells of fetal small intestine either, but this could be due to difficulties in culturing epithelial, polarized cells, which could have interfered with megalin expression. It is therefore unknown at this point whether fetal terminal ileum expresses megalin or not.

Our mRNA‐based results indicate that the *LRP2* expression in human terminal ileum decreases significantly from fetal to adult life. If this is the case for megalin expression as well, it would be in line with a very recent study published by Ilundain and coworkers (Vazquez‐Carretero et al. 2014), who investigated the expression of *lrp2*/megalin, *cubn*/cubilin and *amn*/amnionless in the small intestine of newborn and young rats and found that while the *cubn*/cubilin and *amn*/amnionless expression was maintained after birth, *lrp2*/megalin expression decreased drastically immediately after birth (Vazquez‐Carretero et al. 2014). We speculate that the fetal terminal ileum may serve a ‘kidney‐like’ function. Fetal ileal cubilin, amnionless, and megalin may contribute to the absorption of ligands from amniotic fluid. It is known that the human fetus loses proteins and nutrients into the urine due to immature kidney function (Springate et al. 1987; Carmody and Charlton 2013). Furthermore, the fetus drinks amniotic fluid and thereby its own urine (Pitkin and Reynolds 1975). Assuming that cubilin and megalin ligands reaches the fetal terminal ileum and do not get degraded along the gastrointestinal system, as they do in adults (Britton and Koldovsky 1989; Koldovsky et al. 1991), they could be taken up by enterocytes of the terminal ileum – mediated by cubilin, amnionless and/or megalin. This could justify megalin coexpression with cubilin and amnionless in fetal terminal ileum in contrast to its absent expression in adult terminal ileum. At this point, however, with no evidence of megalin expression in human fetal terminal ileum, this remains speculative. Future studies are required to enlighten the expression of cubilin, amnionless and megalin at protein level in fetal terminal ileum to validate this hypothesis. One could furthermore speculate that megalin serves a more general role during fetal development of terminal ileum, and that this function is not required in the adult stage. This would resemble its established role in development of the kidney proximal tubules (Leheste et al. 1999). Apparently, amnionless does not serve such a role during development of neither the proximal tubules nor the intestines (Strope et al. 2004).

In conclusion, the main findings of this study revealed that previous models designating megalin as an obligatory coreceptor for cubilin in terminal ileum should be revised, since *LRP2*/megalin is not expressed in adult terminal ileum. Our data advocate that cubilin and amnionless expressed in adult human terminal ileum act independently of megalin during B_12_‐absorption. Accordingly, the necessity of megalin for cubilin function should be considered as tissue and/or ligand specific.

## Acknowledgments

We thank Gitte Fynbo Biller, Mette Singers Johansen, and Jeanette Georgsen for technical assistance, S. K. Moestrup for anti‐cubilin and anti‐megalin antibodies, and Tina Storm, and Tine Kjaergaard for fruitful discussions. The author Mette Madsen has previously published under the name *Mette Kristiansen*. The author Louise Lund Jensen has previously published under the name *Louise Lund Andersen*.

## Conflict of Interest

None declared.
